# Supplementary material for: Leveraging transfer learning for accurate estimation of ionic migration barriers in solids
Source: NPJ Comput Mater. 2026 Feb 2;12(1):88. doi: 10.1038/s41524-026-01972-8 (PMC12904795; doi:10.1038/s41524-026-01972-8)
Supplement: Supplementary file 1 — Supplementary Information [file 41524_2026_1972_MOESM1_ESM.pdf]

–Electronic Supporting Information– Leveraging transfer learning for accurate estimation of ionic migration barriers in solids

Reshma Devi[1], Keith T. Butler[2,*], and Gopalakrishnan Sai Gautam[1,*]

[1]Department of Materials Engineering, Indian Institute of Science, Bengaluru 560012, Karnataka, India
[2]Department of Chemistry, University College London, London WC1E 6BT, United Kingdom
[*]k.t.butler@ucl.ac.uk; saigautamg@iisc.ac.in

S1 Comparison of calculated barriers against experimental migration enthalpies

Nudged elastic band (NEB) calculated migration barriers correspond to the 0 K internal energy barrier (i.e., E_m) at fixed lattice parameter and, by construction, excludes the $p-V$ contributions that are typically 'small' in solids, especially in frameworks that have low isothermal compressibilities. Thus, it is often assumed that the experimental migration enthalpy $(h_m) \approx E_m$.

To assess the validity of the $E_m \approx h_m$ approximation, we compare NEB-calculated E_m with literature experimental h_m for 15 compounds in Figure S1. Specifically, we consider $LiVPO_4F$,[1] $LiCoO_2$,[2] $LiFePO_4$,[3–5] $Mg_{0\cdot148}Ti_2S_4$,[6] $MgSc_2Se_4$,[7] Na_3PS_4,[8] $LiMn_2O_4$,[9] $LiCoPO_4$,[10] O3-$NaCoO_2$,[11] $Li_{10}GeP_2S_{12}$,[12] Na_3PSe_4,[13] $Na_3V_2(PO_4)_3$,[14] $LiGe_2(PO_4)_3$,[15] d$-LiV_2O_5$,[16] and $Li_7P_3S_{11}$[17]. The experimental h_m are from temperature-dependent galvanostatic intermittent titration technique or electronic impedance spectroscopy measurements, where the Arrhenius slope of the diffusivity yields (h_m) and thus implicitly includes $(p-V)$ effects. Eight of the 15 points lie 'close' to the parity line (dashed black line), indicating that E_m is a reliable approximation for h_m in many cases. The largest deviations (Na_3PSe_4 and Na_3PS_4) stem from experimental "activation energies" that include defect-formation contributions in addition to migration. The remaining discrepancies are primarily attributable to known limitations of the exchange–correlation functionals used in the density functional theory based E_m estimates.[18]

S2 Default model configuration and other hyperparmeters

The default atomistic line graph neural network (ALIGNN) model configuration utilized for the study is listed in Table S1. We maintained the number of epochs at 500 for the training of all four models, as we consistently observed the validation loss converging around this epoch count in all instances. The default batch size was set to 16 for all the scratch and modified multi-property pre-trained (MPT) models during the initial performance analysis. The optimal batch size for the best-performing model (MODEL-3) and the model with attention blocks (MODEL-4) were further tuned and the batch size that offered the best test score is reported in Table S1. The architecture of the MPT model is visualized in Figure S2.

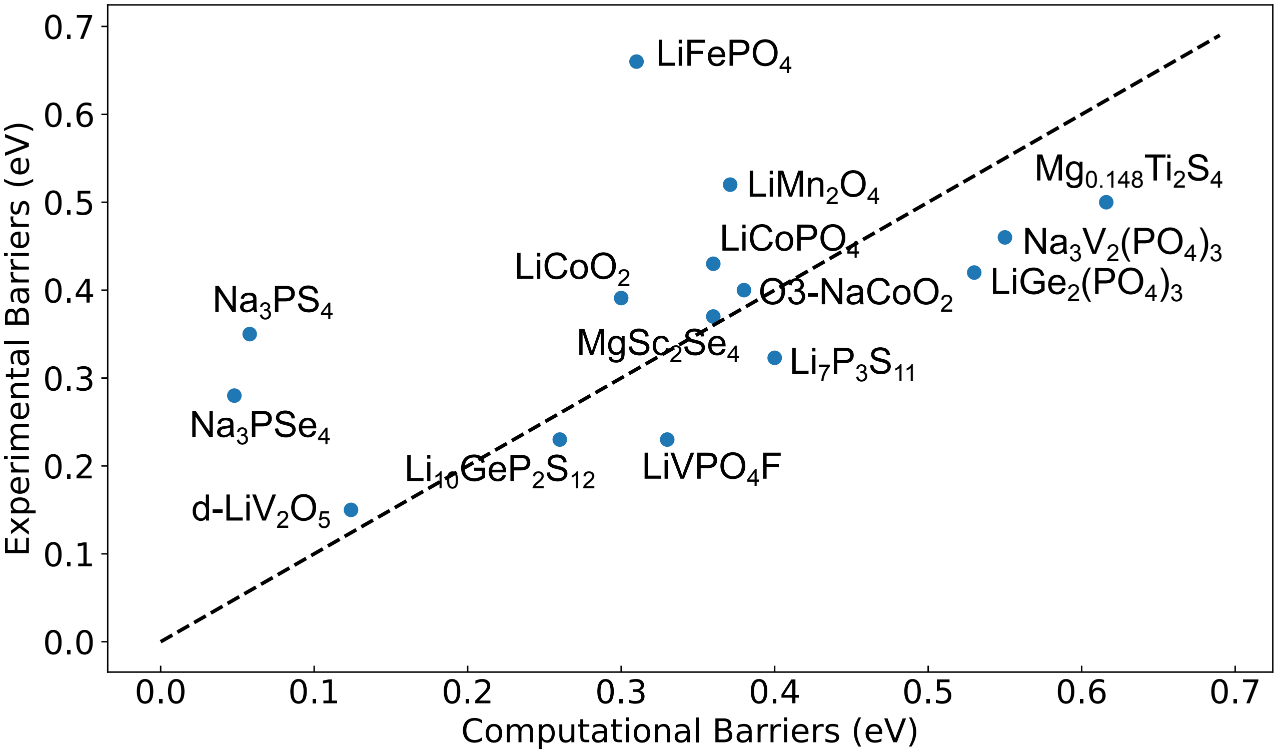

Figure S1. Comparison of experimental h_m and computational E_m. A comparison of the experimentally and computationally reported migration barriers for 15 different systems, that are a part of the overall dataset used in this work.

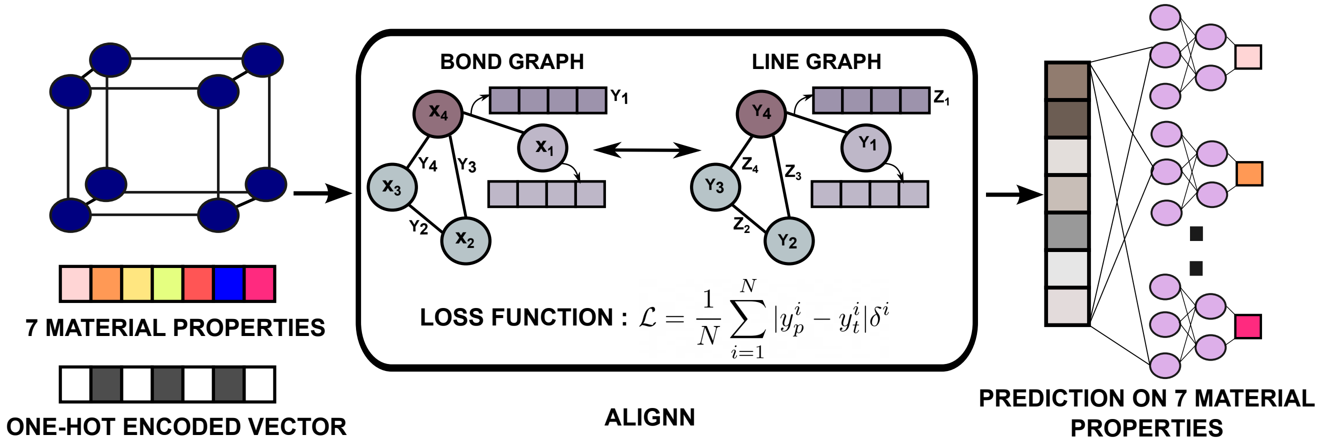

Figure S2. Architecture of the MPT model. The final multi-layer perceptron layer of ALIGNN is modified such that there are seven prediction heads corresponding to each of the seven properties in the cumulative dataset. The model takes both the property and the one-hot encoded vector and is trained using a modified loss function, as depicted within the central panel of the figure.

**Parameter**	**Value**
ALIGNN layers	4
Graph convolutional network layers	4
Atom input features	92
Edge input features	80
Bond angle input features	40
Embedding features	64
Hidden features	256
Normalization	Batch
Default Batch size	16
Batch size for MODEL-3	128
Batch size for MODEL-4	64
Pre-training (PT) learning rate	0.0001
Fine-tuning (FT) learning rate	0.001
Optimizer	Adaptive moment estimation with weight decay
Weight decay	0.00001
Random seed	123
Epochs	500

Table S1. Configuration of the ALIGNN model used in this work.

S3 Construction of classical machine learning models

The features used in the classical machine learning models, namely random forest regression (RFR) and gradient boosted regression (GBR), were obtained primarily from two sources: the matminer.features module within the matminer library, and a collection of nine manually engineered features designed to describe characteristics of cation migration pathways. The matminer library can comprehensively generate numerical descriptors from materials data, covering a broad spectrum of features, including elemental features (derived from properties such as atomic mass, electronegativity, and ionization energy), stoichiometric characteristics, valence electron configurations, statistical features representing the local atomic environment, crystal graph-based descriptors, Coulombic interactions, and radial distribution function-based descriptors. The manually engineered features encompassed the average bond length of the migrating cation at both its initial and final positions, the total migration path distance, and the coordination number of the migrating cation. Additionally, Voronoi polyhedra were constructed around each site occupied by the migrating cation. From these polyhedra, several metrics were calculated, including the average solid angle subtended by each polyhedral face, average facet area, facet distance, the number of facet vertices, and polyhedral volume.

Starting from an initial pool of 277 features, Pearson's correlation coefficient was applied to identify the most informative features. We excluded features showing high inter-correlation (correlation coefficient > 0.95) and retained only those demonstrating at least a modest correlation (≥ 0.10) with the target variable. We chose a relatively low correlation threshold for discarding features since the overall correlation observed between all features and the target variable is low. Our filtering strategy ultimately led to a refined, final feature set consisting of 75 features. The selected 75 (with 5 manually engineered features) features with their brief descriptions are listed in Table S2. Note that the statistics (mean, median, mode, standard deviation, maxima, minima, and range) are obtained considering all the elements forming the respective compounds. The hyperparameters used for the construction of RFR and GBR are listed in Table S3.

Table S2. Selected matminer-derived and manually engineered features and their descriptions

Index	Feature Name	Description
**Matminer features**		
1	MagpieData mode Number	Mode of the atomic number
2	MagpieData minimum MendeleevNumber	Minimum Mendeleev number
3	MagpieData mean MendeleevNumber	Mean Mendeleev number
4	MagpieData avg_dev MendeleevNumber	Average deviation of Mendeleev number
5	MagpieData minimum MeltingT	Minimum melting temperature (K)
6	MagpieData maximum MeltingT	Maximum melting temperature (K)
7	MagpieData avg_dev MeltingT	Average deviation of melting temperature (K)
8	MagpieData mode MeltingT	Mode of the melting temperature (K)
9	MagpieData mean Column	Mean group number (column) in the periodic table
10	MagpieData avg_dev Column	Average deviation of group number in the periodic table
11	MagpieData minimum Row	Minimum row number in the periodic table
12	MagpieData range Row	Range of row numbers in the periodic table
13	MagpieData avg_dev Row	Average deviation of row numbers in the periodic table
14	MagpieData range CovalentRadius	Range of covalent radii (pm)
15	MagpieData mean CovalentRadius	Mean covalent radius (pm)

Continued on next page

Table S2: (continued)

Index	Feature Name	Description
16	MagpieData avg_dev CovalentRadius	Average deviation of covalent radius (pm)
17	MagpieData maximum Electronegativity	Maximum electronegativity
18	MagpieData range Electronegativity	Range of electronegativity
19	MagpieData mean Electronegativity	Mean electronegativity
20	MagpieData avg_dev Electronegativity	Average deviation of electronegativity
21	MagpieData mode Electronegativity	Mode of electronegativity
22	MagpieData minimum NsValence	Minimum number of s-valence electrons
23	MagpieData range NsValence	Range of number of s-valence electrons
24	MagpieData mean NsValence	Mean number of s-valence electrons
25	MagpieData avg_dev NsValence	Average deviation of number of s-valence electrons
26	MagpieData mean NpValence	Mean number of p-valence electrons
27	MagpieData mode NdValence	Mode of the number of d-valence electrons
28	MagpieData minimum NValence	Minimum total number of valence electrons
29	MagpieData range NValence	Range of total number of valence electrons
30	MagpieData avg_dev NValence	Average deviation of total number of valence electrons
31	MagpieData avg_dev NpUnfilled	Average deviation of number of unfilled p-orbitals
32	MagpieData mode NpUnfilled	Mode of the number of unfilled p-orbitals
33	MagpieData minimum NUnfilled	Minimum number of unfilled orbitals
34	MagpieData mean NUnfilled	Mean number of unfilled orbitals
35	MagpieData mode NUnfilled	Mode of the number of unfilled orbitals
36	MagpieData minimum ground state (GS) volume_pa	Minimum atomic volume from the GS
37	MagpieData mean GS volume_pa	Mean atomic volume from the GS
38	MagpieData mode GS volume_pa	Mode of the atomic volume from the GS
39	mean CN_VoronoiNN	Mean coordination number using Voronoi Nearest Neighbors
40	avg_dev CN_VoronoiNN	Average deviation of coordination number using Voronoi Nearest Neighbors
41	max relative bond length	Maximum relative bond length in the structure
42	maximum neighbor distance variation	Maximum variation in neighbor distances
43	mean neighbor distance variation	Mean variation in neighbor distances
44	mean ordering parameter shell 1	Mean ordering parameter for the first coordination shell
45	mean ordering parameter shell 2	Mean ordering parameter for the second coordination shell

Continued on next page

Table S2: (continued)

Index	Feature Name	Description
46	mean local difference in MendeleevNumber	Mean of local differences in Mendeleev number
47	minimum local difference in MeltingT	Minimum of local differences in melting temperature
48	range local difference in MeltingT	Range of local differences in melting temperature
49	mean local difference in MeltingT	Mean of local differences in melting temperature
50	avg_dev local difference in MeltingT	Average deviation of local differences in melting temperature
51	mean local difference in Column	Mean of local differences in column number
52	minimum local difference in Row	Minimum of local differences in row number
53	maximum local difference in Row	Maximum of local differences in row number
54	mean local difference in Row	Mean of local differences in row number
55	range local difference in CovalentRadius	Range of local differences in covalent radius
56	mean local difference in CovalentRadius	Mean of local differences in covalent radius
57	avg_dev local difference in CovalentRadius	Average deviation of local differences in covalent radius
58	range local difference in Electronegativity	Range of local differences in electronegativity
59	avg_dev local difference in Electronegativity	Average deviation of local differences in electronegativity
60	avg_dev local difference in NsValence	Average deviation of local differences in s-valence electrons
61	maximum local difference in NpValence	Maximum of local differences in p-valence electrons
62	avg_dev local difference in NpValence	Average deviation of local differences in p-valence electrons
63	range local difference in NdValence	Range of local differences in d-valence electrons
64	avg_dev local difference in NdValence	Average deviation of local differences in d-valence electrons
65	minimum local difference in NValence	Minimum of local differences in total valence electrons
66	maximum local difference in NValence	Maximum of local differences in total valence electrons
67	range local difference in NValence	Range of local differences in total valence electrons
68	avg_dev local difference in NValence	Average deviation of local differences in total valence electrons
69	maximum local difference in NpUnfilled	Maximum of local differences in unfilled p-orbitals
70	maximum local difference in SpaceGroupNumber	Maximum of local differences in space group number
**Manually engineered features**		
71	ini_cn	Coordination number of the intercalating ion
72	avg_ini_solid_angle	Average solid angle of the Voronoi polyhedra

Continued on next page

Table S2: (continued)

Index	Feature Name	Description
73	avg_ini_facet_area	Average facet area
74	n_verts_ini	Average number of vertices per facet
75	path_distance	Path distance

Parameter	Value
n_estimators	150
Random state	123
Cross-fold validation	5

Table S3. Hyperparameters for RFR and GBR.

S4 MACE-NEB E_m Calculations

Multi atomic cluster expansion (MACE) based NEB calculations were performed using the PT foundational model 'MACE-MP-0', which leverages a dataset of density functional theory relaxation trajectories from all compounds within the materials project to accurately predict forces and energies. During the NEB calculations, the Broyden–Fletcher–Goldfarb–Shanno (BFGS) algorithm was used to converge the forces to a threshold of |0.05| eV/Å, with a maximum of 1000 steps allowed for convergence. For MACE-NEB-RELAX migration barrier (E_m) estimation, the endpoints of the migration pathway were relaxed using a quasi-Newton optimizer, again with a force convergence threshold of |0.05| eV/Å. The specific parameters employed for the MACE-NEB calculations are detailed in Table S4.

Parameter	Value
BFGS force threshold	|0.05| eV/Å
Maximum number of convergence steps	1000
Optimizer for endpoint relaxation	quasi-Newton
Optimizer for NEB calculation	BFGS
MACE model	MACE-MP-0
MACE model type	large
Float type	float64

Table S4. MACE-NEB calculation parameters.

S5 Performance metrics

MODELS	Train		Test	
	R^2 score	MAE (eV)	R^2 score	MAE (eV)
RFR	0.837	0.178	0.396±0.295	0.318±0.055
GBR	0.879	0.198	0.097±0.679	0.299±0.073
SCRATCH-ADD	0.575	0.203	-0.035±0.325	0.451±0.067
SCRATCH-SUB	-17.179	2.434	-11.842±4.798	2.623±0.118
SCRATCH-CC	0.777	0.157	-1.241±1.231	0.536±0.111
SCRATCH-MODEL-3	0.981	0.074	-2.414±0.386	0.541±0.065
MODEL-1-ADD	0.533	0.246	-0.076±0.307	0.400±0.074
MODEL-1-SUB	0.165	0.4411	-0.083±0.100	0.462±0.068
MODEL-1-CC	0.562	0.241	0.219±0.148	0.379±0.060
MODEL-1-CC-SINGLE-PATH	0.912	0.107	0.603±0.263	0.249±0.045
MODEL-2	0.579	0.218	0.261±0.348	0.382±0.059
MODEL-3	0.940	0.085	0.703±0.109	0.261±0.034
MODEL-4	0.822	0.171	0.634±0.107	0.277±0.039
MACE-NEB-RELAX	0.099	0.363	0.185±0.601	0.340±0.064

Table S5. Model performance metrics for training and test sets. MAE is the mean absolute error. ADD, SUB, and CC refer to addition, subtraction, and concatenation of embeddings from two MPT model instances. Descriptions of the different model architectures are provided in the Methods section of the main text. 'SCRATCH-MODEL-3' refers to the MODEL-3 architecture, i.e., structure with a five-image band input, that is trained from scratch directly on the train dataset. The standard errors reported for the test set for all models are obtained from the bootstrapped test set.

S6 Parity plots

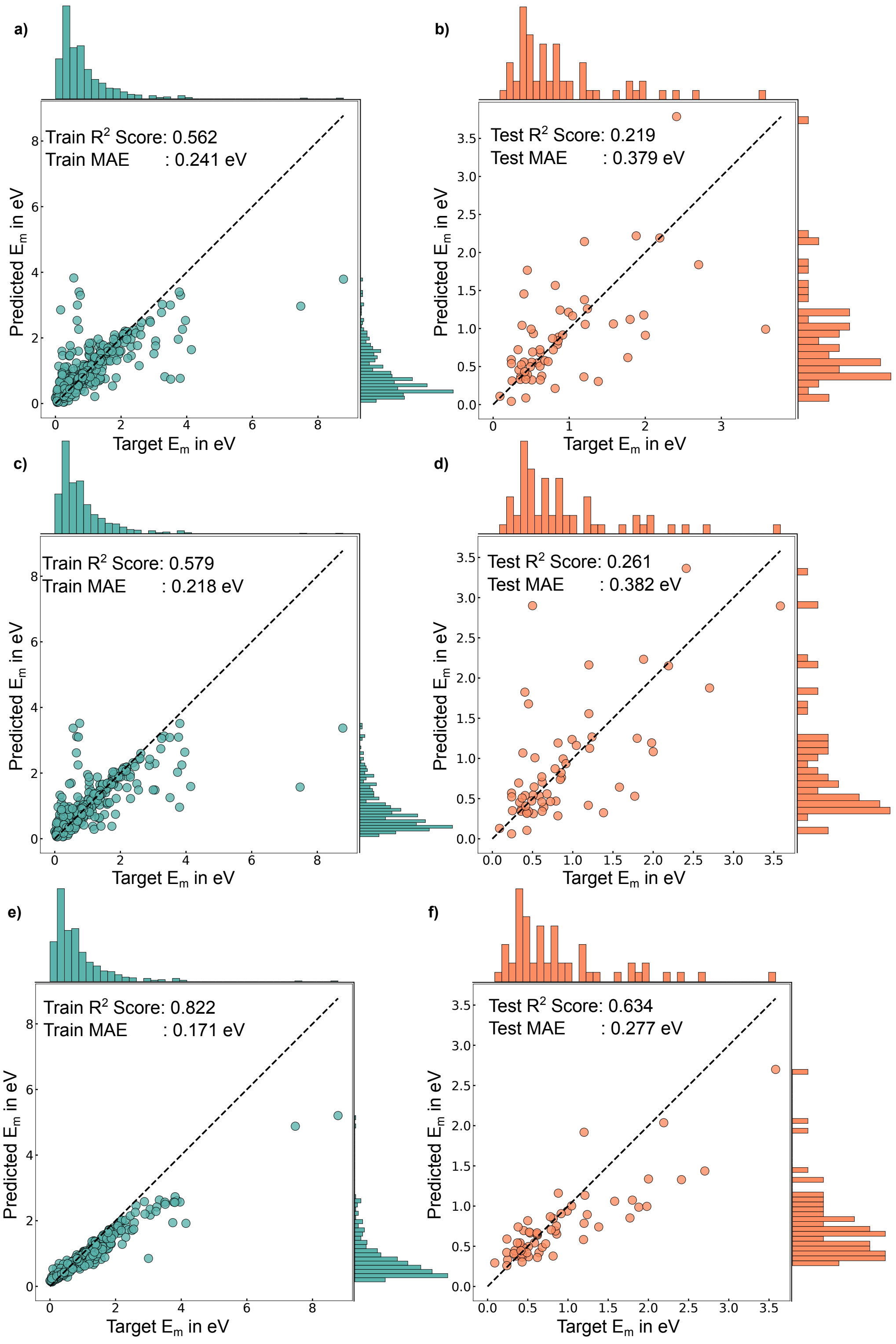

Figure S3. Prediction vs. target for MODEL-1, MODEL-2, and MODEL-4 . Parity plot illustrating the prediction versus the target for training and test dataset for MODEL-1 (panels a and b), MODEL-2 (panels c and d), and MODEL-4 (panels e and f). The histograms on the right and top margins correspond to the frequency distribution of prediction and target E_m, respectively.

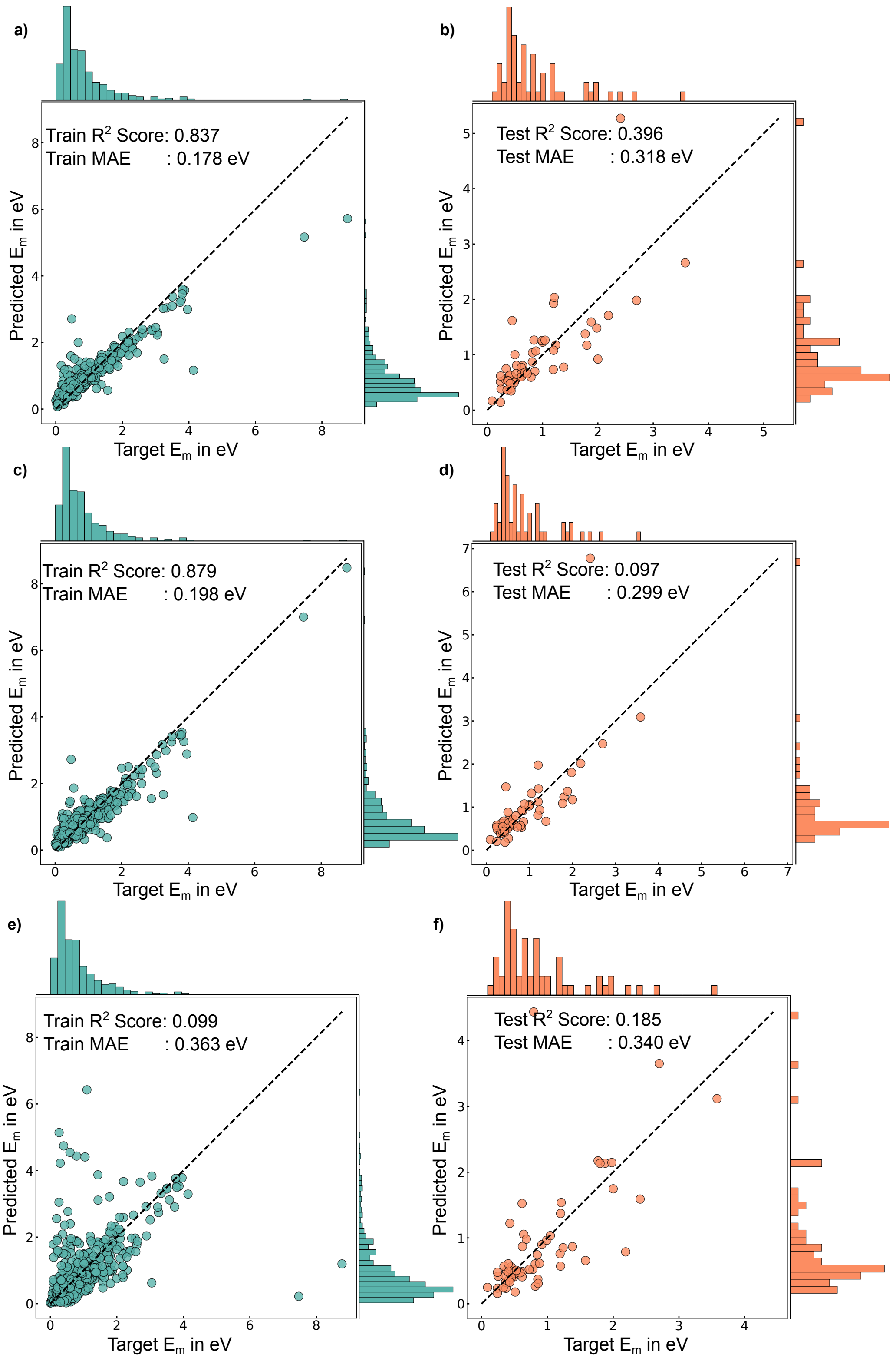

Figure S4. **Prediction vs target for RFR, GBR, and MACE-NEB-RELAX**. Parity plot illustrating the prediction versus the target for training and test dataset for RFR (panels a and b), GBR (panels c and d), and MACE-NEB-RELAX (panels e and f). Notations used are identical to Figure S3.

S7 Dataset size influence in MODEL-4

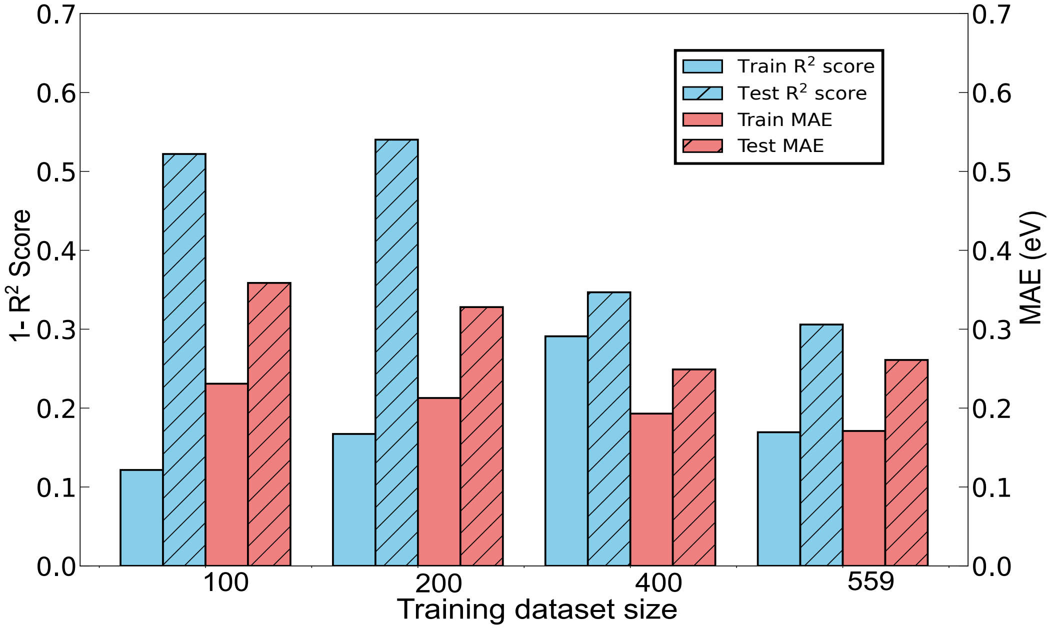

Figure S5. **Performance of MODEL-4 as a function of training dataset size**. The performance metrics, namely 1-R^2 scores (blue bars) and MAEs (red bars) for MODEL-4 are plotted for four different training dataset sizes. Note that the performance of MODEL-4, as quantified using test R^2 scores and MAEs, improves as the training dataset size increases.

References

1. Xiao, P., Lai, M. & Lu, L. Transport and electrochemical properties of high potential tavorite livpo4f. *Solid State Ionics* **242**, 10–19 (2013).
2. Qiu, X.-Y. *et al.* Electrochemical and electronic properties of licoo 2 cathode investigated by galvanostatic cycling and eis. *Phys. chemistry chemical physics* **14**, 2617–2630 (2012).
3. Schmidt, J. P. *et al.* Studies on $LiFePO_4$ as Cathode Material Using Impedance Spectroscopy. *J. Power Sources* **196**, 5342–5348 (2011).
4. Takahashi, M., Tobishima, S.-i., Takei, K. & Sakurai, Y. Reaction behavior of lifepo4 as a cathode material for rechargeable lithium batteries. *Solid state ionics* **148**, 283–289 (2002).
5. Maier, J. & Amin, R. Defect chemistry of lifepo4. *J. The Electrochem. Soc.* **155**, A339 (2008).
6. Sun, X. *et al.* A High Capacity Thiospinel Cathode for Mg Batteries. *Energy Environ. Sci.* **9**, 2273–2277 (2016).
7. Canepa, P. *et al.* High Magnesium Mobility in Ternary Spinel Chalcogenides. *Nat. Commun* **8**, 1–8 (2017).
8. Moon, C. K. *et al.* Vacancy-Driven Na^+ Superionic Conduction in New Ca-doped Na_3PS_4 for All-Solid-State Na-ion Batteries. *ACS Energy Lett.* **3**, 2504–2512 (2018).
9. Kuwata, N., Nakane, M., Miyazaki, T., Mitsuishi, K. & Kawamura, J. Lithium diffusion coefficient in limn2o4 thin films measured by secondary ion mass spectrometry with ion-exchange method. *Solid State Ionics* **320**, 266–271 (2018).
10. Prabu, M., Selvasekarapandian, S., Reddy, M. & Chowdari, B. Impedance studies on the 5-v cathode material, licopo4. *J. Solid State Electrochem.* **16**, 1833–1839 (2012).
11. Shibata, T., Fukuzumi, Y., Kobayashi, W. & Moritomo, Y. Fast discharge process of layered cobalt oxides due to high na+ diffusion. *Sci. reports* **5**, 9006 (2015).
12. Kamaya, N. *et al.* A lithium superionic conductor. *Nat. materials* **10**, 682–686 (2011).
13. Bo, S.-H., Wang, Y., Kim, J. C., Richards, W. D. & Ceder, G. Computational and experimental investigations of na-ion conduction in cubic na3pse4. *Chem. Mater.* **28**, 252–258 (2016).
14. Wang, Q., Zhang, M., Zhou, C. & Chen, Y. Concerted ion-exchange mechanism for sodium diffusion and its promotion in na3v2 (po4) 3 framework. *The J. Phys. Chem. C* **122**, 16649–16654 (2018).
15. Francisco, B. E., Stoldt, C. R. & M'Peko, J.-C. Energetics of ion transport in nasicon-type electrolytes. *The J. Phys. Chem. C* **119**, 16432–16442 (2015).
16. Vijayakumar, M. & Selvasekarapandian, S. Transport properties of lixv2o5 (x= 0.6–1.6) electrode material. *Mater. research bulletin* **38**, 1735–1743 (2003).
17. Xu, R. *et al.* Preparation of li7p3s11 glass-ceramic electrolyte by dissolution-evaporation method for all-solid-state lithium ion batteries. *Electrochimica Acta* **219**, 235–240 (2016).
18. Devi, R., Singh, B., Canepa, P. & Sai Gautam, G. Effect of exchange-correlation functionals on the estimation of migration barriers in battery materials. *npj Comput. Mater.* **8**, 160 (2022).
